# Thermal Properties of Polysiloxane/Ag Nanocomposites with Different Network Structures and Distributions of Si–H Groups

**DOI:** 10.3390/ma17235809

**Published:** 2024-11-27

**Authors:** Monika Wójcik-Bania, Edyta Stochmal

**Affiliations:** 1 Faculty of Geology, Geophysics, and Environmental Protection, AGH University of Krakow, 30-059 Kraków, Poland; 2 Faculty of Materials Science and Ceramics, AGH University of Krakow, 30-059 Kraków, Poland; stochmal@agh.edu.pl

**Keywords:** polysiloxanes, polysiloxane networks, silver nanoparticles, nanocomposites, thermal properties, silicone oxycarbide

## Abstract

Polysiloxanes with silver nanoparticles (Ag NPs) have garnered attention for their distinctive physicochemical properties, which make them promising candidates for advanced material applications. This study presents a systematic investigation into the thermal properties and degradation mechanisms of polysiloxane/Ag nanocomposites, emphasising the innovative incorporation of Ag NPs directly into polysiloxane networks via in situ reduction of Ag⁺ ions by Si-H groups. Six polysiloxane matrices were synthesised by hydrosilylation of poly(methylhydrosiloxane) (PMHS) or poly(vinylsiloxane) (polymer V_3_) with three cross-linking agents of varying molecular structures and functionality. Thermogravimetric analysis combined with mass spectrometry revealed that the introduction of Ag NPs alters the thermal properties of polysiloxane networks, primarily affecting the redistribution of Si bonds that occurs during the pyrolysis of these systems. Monitoring the pyrolysis process using FTIR spectroscopy allowed us to investigate the effect of the presence of Ag NPs on the degradation mechanism of the studied nanocomposites. The presence of the free-carbon phase and metallic silver phase in the Ag-containing silicon oxycarbide materials obtained was confirmed by Raman spectroscopy and XRD analyses, respectively. These findings demonstrate the possibility of fabricating Ag/SiOC materials with ceramic residues in the range of 43 to 84%. This work provides new insights into the thermal behaviour of polysiloxane/Ag nanocomposites and underscores their potential for high-performance applications in thermally demanding environments.

## 1. Introduction

Advanced nanocomposite materials based on polymers are gaining significant attention due to their potential to enhance the mechanical and physicochemical properties of polymer matrices, thus expanding their applicability in various fields [1]. Among these, polysiloxane-based nanocomposites have emerged as a particularly promising class of materials for advanced applications [2,3,4,5]. Polysiloxanes, often referred to as silicones, are unique polymers characterised by repeating silicon–oxygen (-Si-O-) units and organic groups bonded to silicon atoms. This distinctive siloxane structure imparts remarkable properties such as high thermal stability, hydrophobicity, elasticity, and chemical resistance, which are not typically observed in other polymer types [6,7]. These properties make polysiloxanes suitable for a multitude of applications, ranging from aerospace and automotive industries to biomedical fields, wherein they may be employed in coatings, adhesives, and flexible components. The increasing demand for high-performance materials across various industries is driving intensive research into polysiloxane-based nanocomposites, into which the incorporation of numerous nanoadditives is creating new possibilities and advancing material science.

One of the most promising types of nanoadditives incorporated into polysiloxane matrices is metal nanoparticles, particularly silver nanoparticles (Ag NPs), which are classified as zero-dimensional (0D) nanofillers [3,5]. Ag NPs can be incorporated into polysiloxane matrices through various synthesis methods, for example by silver salt reduction [8], metal vapour synthesis [9], or plasma polymerisation with simultaneous silver sputtering [10]. Ag NPs incorporated into polysiloxane matrices offer new possibilities for the use of these nanocomposites. An example is the potent antibacterial activity of the polysiloxane/Ag nanocomposite, which has shown efficacy against both *Escherichia coli* and *Staphylococcus aureus*, two common pathogens associated with healthcare-related infections [11,12]. This antimicrobial effect, combined with improved cell adhesion and biocompatibility [13,14], underscores the potential of these nanocomposites as advanced materials in medical applications, such as antimicrobial coatings for medical devices and wound dressings, for which both sterility and biocompatibility are crucial. Ag NPs incorporated into polydimethylsiloxane (PDMS), can create tunable fibre optical cavities with linearity and high sensitivity in a wide temperature range, making these new nanocomposites a very good candidate for application as an optical sensing element [15]. Research conducted by Masraff et al. demonstrated that alignment of Ag NPs within polydimethylsiloxane under a direct current electric field significantly improves its thermal conductivity by an average of 95% in the longitudinal direction [16]. Another example of applying polysiloxane/Ag nanocomposites is their potential as an eco-friendly antifouling coating in the maritime industry, utilising superhydrophobic properties to prevent biofouling adhesion and enhance surface durability without releasing toxic substances [17].

Ag NPs also serve as highly effective catalysts, attributed to their high surface area and distinctive surface properties that enhance catalytic performance. They have been successfully applied in a variety of organic transformations, particularly to facilitate the formation of key bonds, such as C-C, C-N, C-S, and C-O, in conjunction with their role in essential reduction and oxidation reactions, demonstrating their versatility and efficiency across in various catalytic applications [18,19]. Ag NPs have also been successfully used for the reduction of hazardous dyes [20,21].

In recent years, research has been conducted to develop heterogeneous catalysts based on Ag NPs embedded in various polymer matrices. Examples include nanocomposites with Ag NPs supported on chitosan [22], poly(acrylic acid) hydrogels [22], polypyrrole [23], and polystyrene microspheres [24]. Despite the remarkable properties of polysiloxanes, their applications as supports for Ag NPs and their study as heterogeneous catalysts have been relatively underexplored.

In our previous work, we demonstrated that silver ions could be directly reduced in a polysiloxane matrix by using Si-H groups present in the system, which exhibited reducing properties [25]. The resulting polysiloxane/Ag nanocomposites showed high catalytic efficiency in the methyl red degradation reaction and retained their performance in five reaction cycles, indicating their stability and reusability. It is worth mentioning that polysiloxanes are precursors of silicon oxycarbides (SiOC), analogues of amorphous silica in which some of the divalent oxygen atoms have been replaced by tetravalent carbon atoms [26]. SiOC materials offer advantages over polysiloxanes, including higher thermal stability, enhanced chemical resistance, and better mechanical properties that make them suitable for extreme conditions, which improve their effectiveness in catalytic applications. Recent advances in SiOC materials have demonstrated their potential for biomedical applications [27]. For example, Ag/SiOC plasma polymer coatings [28] and Ag/SiOC composite fibres produced by electrospinning [29] have demonstrated excellent antimicrobial activity against various bacterial strains. However, the integration of Ag NPs within SiOC matrices remains underexplored, particularly in the context of their potential catalytic applications. This study provides new insights into these areas by leveraging the reducing properties of Si-H groups in polysiloxane networks to directly embed Ag NPs in polymer precursors of SiOC materials.

Unlike previous studies that predominantly focused on the antimicrobial activity and applications of polysiloxane/Ag nanocomposites, this work systematically investigates their thermal behaviour and degradation mechanisms, areas that have received limited attention. Specifically, the role of the network structure and the distribution of the Si-H group on the thermal stability and degradation pathways of polysiloxane/Ag nanocomposites has not been thoroughly addressed. This study bridges this gap by demonstrating that the incorporation of Ag NPs into polysiloxane networks can significantly influence the bond redistribution during thermolysis and the ceramic yield of Ag/SiOC materials. To achieve this, the effect of Ag NPs on the thermal properties of six polysiloxane networks with different structures and distributions of Si-H groups was examined, focusing on degradation mechanisms and thermal behaviour using techniques such as thermogravimetry coupled with mass spectrometry and FTIR spectroscopy. The obtained Ag/SiOC nanocomposites were further characterised through Raman spectroscopy, SEM imaging, and XRD analysis.

The insights gained from this study could pave the way for the development of highly efficient, thermally and chemically stable catalysts for industrial organic transformations. While this work focuses on polysiloxane networks with specific structures and Si-H group distributions, future research could explore the effects of alternative cross-linking agents or functionalised Ag NPs to further tailor thermal and catalytic properties.

## 2. Materials and Methods

### 2.1. Materials

The following substances were purchased from ABCR (Karlsruhe, Germany): PMHS (viscosity 35–45 cSt) with trimethylsiloxy groups at both ends; vinylsiloxanes (1,3-divinyltetramethyldisiloxane (^Vi^MM^Vi^), 1,3,5,7-tetramethyl-1,3,5,7-tetravinylcyclotri-siloxane (D_4_^Vi^), tetrakis(vinyldimethylsiloxy)silane (QM^Vi^_4_)); hydrosiloxanes (1,1,3,3-tetramethyldisiloxane (^H^MM^H^); 2,4,6,8-tetramethylcyclotetrasiloxane (D_4_^H^); tetrakis(dimethylsiloxy)silane (Q(M^H^)_4_)); and a catalyst (platinum(0)-1,3-divinyl-1,1,3,3-tetramethyldisiloxane complex (Karstedt catalyst) solution in xylene (2 wt.% Pt)). Silver heptafluorobutyrate 97% (CF_3_CF_2_CF_2_COOAg) was provided by Sigma-Aldrich (Poznań, Poland). The V_3_ polymer was synthesised using kinetically controlled anionic ring-opening polymerisation of the 1,3,5-trimethyl-1,3,5-trivinylcyclotrisiloxane according to the procedure described in [30].

The cross-linked systems were prepared using a hydrosilylation reaction with an excess of Si-H groups; the ratio of these groups to vinyl groups was 1.5 to 1. PMHS was cross-linked with three vinyl siloxanes, i.e., ^Vi^MM^Vi^, D_4_^Vi^, and QM^Vi^_4_, while the V_3_ polymer was cross-linked with three hydrosiloxanes, i.e., ^H^MM^H^, D_4_^H^, and Q(M^H^)_4_, according to the procedure described in [31] and [32], respectively. Six polysiloxane networks were obtained, the names of which consist of the symbol of the polymer used and the cross-linking agent; e.g., PMHS(^Vi^MM^Vi^) means PMHS cross-linked with ^Vi^MM^Vi^.

Silver nanoparticles were incorporated into the cross-linked systems from the solution of CF_3_CF_2_CF_2_COOAg in toluene. A detailed description of this process is given in [25]. The samples into which the Ag NPs were introduced contain the Ag symbol in the name, e.g., PMHS(^Vi^MM^Vi^)Ag.

Selected samples were subjected to pyrolysis at temperatures chosen based on DTG curves. The pyrolysis procedure was executed in a quartz tube furnace under an Ar atmosphere. For each trial, a quantity of 0.05 to 0.10 g of the sample under investigation was introduced into the furnace, then heated to the designated temperature at a rate of 5 K/min and held at that temperature for 15 min. Subsequently, gradual cooling to room temperature in an Ar gas flow was carried out in the furnace. The samples after pyrolysis at 1000 °C have the symbol “P” in their name, e.g., PMHS(^Vi^MM^Vi^)_P.

### 2.2. Characterisation Methods

Thermogravimetric (TG) measurements were performed by applying the STA 449 F3 Jupiter, Netzsch (Germany), coupled with a quadrupole mass spectrometer (QMS model 403 C Aeolos, Netzsch, Germany). A sample mass of ca. 10 mg was placed in an alumina crucible and heated from 30 to 1000 °C, at 10 K/min, in flowing helium (50 mL/min). Simultaneous analyses of the evolved gases were carried out using a quadrupole mass spectrometer. Mass spectrometric measurements were conducted using the scan mode across a mass-to-charge (*m/z*) range of 2 to 100, where ‘m’ denotes the mass of the molecule and ‘z’ signifies the charge of the molecule expressed in elementary charge units. The scanning time for each *m/z* was 0.5 s. Thermal patterns were collected and processed using Netzsch Proteus® 6 Thermal Analysis software.

FTIR spectra were collected with the Nicolet 6700 Thermo Scientific spectrometer (Thermo Fisher Scientific, Waltham, MA, USA) using the transmission technique, and the samples were prepared as KBr pellets. Spectral data were collected in the range of 400 to 4000 cm^−1^, with a resolution of 2 cm^–1^, with 64 scans. A quantitative analysis of the FTIR spectra recorded for the pyrolysis products obtained at different temperatures was performed. Calculations were made using the Omnic 8.3 programme after applying the baseline correction to individual spectra. The integral intensity of the band corresponding to Si-H (2160 cm^−1^) and Si-CH_2_-Si (1359 cm^−1^) was calculated and referenced to the Si-CH_3_ band (1260 cm^−1^).

The silver content in the nanocomposites studied before pyrolysis was determined by wavelength-dispersive X-ray fluorescence (XRF) using a Rigaku ZSX Primus II (Rigaku Corporation, Tokyo, Japan) spectrometer with a Rh anode as the X-ray source, and a calibration prepared on the basis of polysiloxane network samples containing silver heptafluorobutyrate as a source of silver [25]. The silver content after pyrolysis at 1000 °C was calculated on the basis of the metal content determined by XRF and the mass loss from thermogravimetric analysis. In the calculations, it was assumed that there was no loss of metal content from the sample during the thermal decomposition of the polysiloxane network.

Powder X-ray diffraction (XRD) patterns were recorded with a SmartLab 9.0 diffractometer (Rigaku Corporation, Tokyo, Japan), equipped with a D/teX Ultra 250 silicon strip detector, applying Ni-filtered Cu Kα (λ = 1.5406 Å) radiation. The data collection range was 2 to 75°2θ with a constant step equal to 0.05°2θ. The samples were measured in a nonrefection holder.

Raman spectra were obtained using a Thermo Scientific DXR Raman microscope (Waltham, MA, USA) with a λ = 532 nm excitation wavelength. Measurements were carried out under the following conditions: laser power of 10 mW, exposure time of 9 s, and 100 repetitions per measurement. The resolution was set at 2 cm⁻^1^. After applying baseline correction, the spectra were decomposed into individual peaks within the 1750–1000 cm⁻^1^ range using Omnic 8.3 software. The experimental data were modelled using a combination of Gaussian and Lorentzian functions. From the Raman spectra showing the D and G bands, the in-plane crystalline size of the graphite domains (L_a_) was calculated. The size of the C domain was determined using the relation proposed by Matthews et al. [33]:

$$L_{a}=C{\left(\frac{I_{D}}{I_{G}}\right)}^{-1}$$

where C is the scaling coefficient and *I_D_/I_G_* is the ratio of the integral intensities of the D and G bands. The ratio of integral intensity of the D and G bands decreased as the laser energy increased, resulting in a variation of the scaling coefficient C with the laser wavelength. To determine the suitable C(λ_L_) value for the excitation line at λ = 532 nm, the approximation C(λ_L_) ≈ C_0_ + λ_L_C_1_ was used. The estimated values for C_0_ and C_1_ were found to be −12.6 nm and 0.033, respectively.

Scanning electron microscopy (SEM) observations were conducted using a Quanta 200 FEG microscope (FEI, Hillsboro, OR, USA) equipped with an X-ray energy-dispersive spectroscopy (EDS) system, as well as secondary electron (SE) and backscattered electron (BSE) detectors. The SEM images presented in this paper were generated by superimposing SE and BSE images.

## 3. Results and Discussion

**Figure 1:**
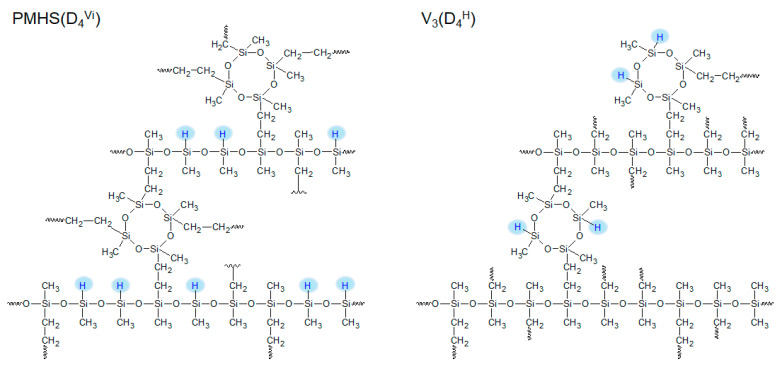
Structure of polysiloxane networks with the same architecture and different arrangement of Si-H groups.

The polysiloxane networks investigated in the present study were obtained as a result of the hydrosilylation of two polymers, i.e., polymer V_3_ and PMHS, with one of three cross-linking agents. In polymer V_3_, there was one vinyl group at each silicon atom, while in PMHS there was a hydrogen atom instead of a vinyl group. The cross-linking agents were hydrogen- and vinylsiloxanes with different molecular structures and numbers of functional groups: difunctional linear ^H^MM^H^/^Vi^MM^Vi^, cyclic tetrafunctional D_4_^H^/D_4_^Vi^, and linear branched tetrafunctional Q(M^H^)_4_/Q(M^Vi^)_4_. The obtained polysiloxane networks had the same network structure but differed in the arrangement of Si-H groups in the polymer matrix. Figure 1 shows the schemes of the polysiloxane networks obtained from the V_3_ and PMHS polymers, which were cross-linked with the cyclic curing agents D_4_^H^ and D_4_^Vi^, respectively.

**Figure 2:**
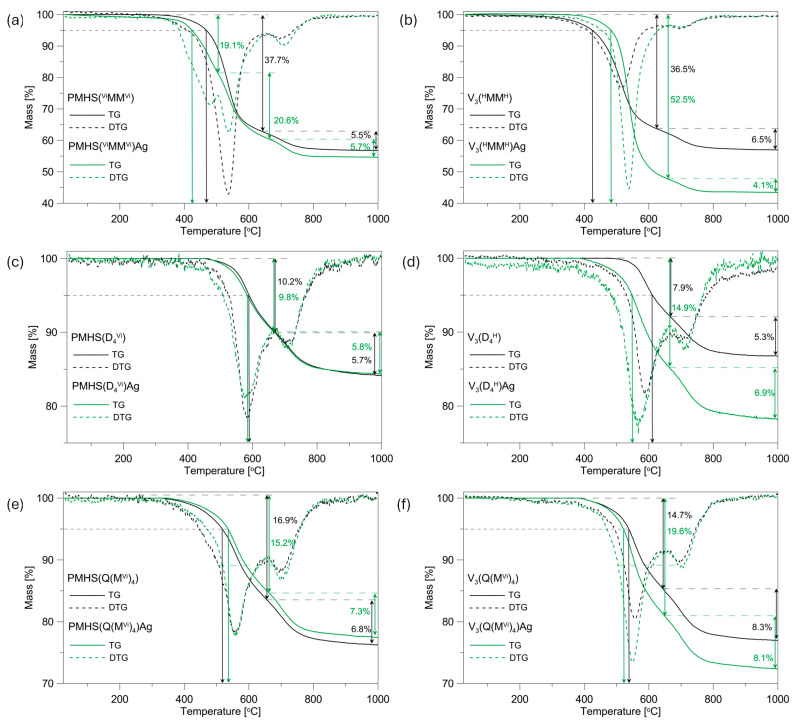
TG and DTG curves of the initial PMHS and V_3_ networks and systems containing Ag NPs: (**a**) PMHS(^Vi^MM^Vi^) and PMHS(^Vi^MM^Vi^)Ag, (**b**) V_3_(^H^MM^H^) and V_3_(^H^MM^H^)Ag, (**c**) PMHS(D_4_^Vi^) and PMHS(D_4_^Vi^)Ag, (**d**) V_3_(D_4_^H^) and V_3_(D_4_^H^)Ag, (**e**) PMHS(Q(M^Vi^)_4_) and PMHS(Q(M^Vi^)_4_)Ag, (**f**) V_3_(Q(M^H^)_4_) and V_3_(Q(M^H^)_4_)Ag.

The thermal characteristics of the initial polysiloxane networks and systems containing Ag NPs were analysed through thermogravimetry (TG). Subsequently, derivative curves (DTG) were generated based on the acquired data, as illustrated in Figure 2. The data extracted from the TG/DTG curves, including residual mass at 1000 °C, peak temperatures, and corresponding mass losses at each degradation stage, are detailed in Table 1.

The type of polymer precursor used has an effect on the thermal properties of the networks studied. When the temperature that causes a mass loss of 5% in the samples analysed (T_5%_) is used as a criterion for thermal stability (Figure 2, Table 1), it can be observed that the PMHS(^Vi^MM^Vi^) system is characterised by a higher thermal stability at 43 °C than its analogue obtained from the V_3_ polymer, while the ceramic residue of these samples is at a similar level and amounts to approximately 57%. The higher thermal stability of the PMHS(^Vi^MM^Vi^) network is probably related to the higher amount of polymer in the system compared to the amount of cross-linking agent. It should be noted that the polymer networks were obtained with a ratio of Si-H groups to Si-CH=CH_2_ equal to 1.5. In the PMHS system, an excess of polymer was used as a source of Si-H groups. The applied difunctional linear cross-linking agent is characterised by a lower thermal stability compared to that of the PMHS. The higher share of the crosslinking agent in the V_3_(^H^MM^H^) polymer network affects the deterioration of its thermal stability in relation to the PMHS(^Vi^MM^Vi^) system. The lower thermal stability of the V_3_(^H^MM^H^) system also affects the temperature of maximum mass loss. The difference in T_max_ 30–650 °C and T_max_ 650–1000 °C for the PMHS(^Vi^MM^Vi^) and V_3_(^H^MM^H^)systems is 23 and 10 °C, respectively.

In the remaining systems, the networks obtained from the V_3_ polymer are characterised by a better thermal stability by approximately 20 °C and a slightly better ceramic residue than their analogues obtained from PMHS, of 2.8% for the D_4_^Vi^/D_4_^H^ systems and 0.7% for the Q(M^Vi^)_4_/Q(M^H^)_4_ systems, respectively. For the systems discussed, only a few degrees of difference in the temperatures of maximum mass losses are observable. The applied tetrafunctional cross-linkers, i.e., cyclic and branched, are characterised by a better thermal stability than linear difunctional ones, and their greater share in the polymer matrix does not have such a great effect on the thermal stability of the studied networks. The slightly better thermal properties of these systems may be influenced by the longer V_3_ polymer chain than that of PMHS. Previous studies have shown that polysiloxane networks obtained from polymers with a larger molecular mass are characterised by better thermal properties [34].

The TG curves that were recorded revealed that the incorporation of Ag NPS into the polysiloxane matrix resulted in an adverse effect on its thermal characteristics in most of the systems studied. The presence of Ag NPs had a detrimental effect on the thermal stability of the four examined polysiloxane networks. An increase in thermal stability was observed only in the PMHS(Q(M^Vi^)_4_)Ag and V_3_(^H^MM^H^)Ag systems, with increases of 19 and 58 °C, respectively. The greatest deterioration of thermal stability by 63 °C was recorded for the V_3_(D_4_^H^)Ag sample, whereas for the PMHS(D_4_^Vi^) system with and without metal, the TG curves were similar, and a slight deterioration in thermal stability of 6 °C was recorded.

The presence of Ag NPs in the studied samples has a greater effect on the thermal properties of the systems obtained from the V_3_ polymer than the PMHS (Figure 2). This is most evident in the context of the ceramic residue after pyrolysis at 1000 °C. In PMHS systems, the differences in the ceramic residue of the initial systems and those after metal incorporation are in the range of 0.3–2.2 wt%. On the other hand, in V_3_ systems, the introduction of metal causes a deterioration of the ceramic residue by 13.6, 8.6, and 4.6% for the V_3_(^H^MM^H^)Ag, V_3_(D_4_^H^)Ag, and V_3_(Q(M^H^)_4_)Ag systems, respectively, compared to the initial networks (Table 1). The greater deterioration of the thermal properties in the V_3_/Ag systems may be related to the cross-linking density of the polysiloxane networks studied and the number of Si-H groups that remained in the systems after metal introduction (the reducing properties of Si-H groups were used for the direct reduction of silver ions in a polysiloxane matrix). Polymer networks obtained from polymer V_3_ were characterised by a lower cross-linking density, that is, a higher average molecular weight between cross-links (M_c_), than systems obtained from PMHS [31,32]. Only PMHS(^Vi^MM^Vi^) and V_3_(^H^MM^H^) systems had a similar M_c_ (approximately 185 g/mol). A lower cross-linking density of the polymer matrix causes Ag NPs to settle not only on their surface, but also in the sample volume during their introduction into the polymer carrier. It should be noted that the metal was introduced in a toluene solution, in which polysiloxane networks swell. The presence of Ag NPs inside the polymer network may limit thermal cross-linking, and thus affect the deterioration of the thermal properties of the systems studied. Furthermore, in V_3_ systems, a greater loss of Si-H groups was observed during the Ag NP introduction process than in PMHS systems [25], which may also have affected the limitation of the thermal cross-linking process in V_3_/Ag systems. It is worth noting the minor differences observed in the thermal properties of the PMHS samples cross-linked with tetrafunctional cross-linking agents after the introduction of Ag NPs. The TG curves of the PMHS(D_4_^Vi^) system, with and without the presence of metal, are nearly identical, and the differences in ceramic yield are only 0.3%. On the other hand, the PMHS(Q(M^Vi^)_4_)Ag system exhibits slightly improved thermal properties compared to the initial polymer network, with a ceramic yield 1.2% higher. These polymer networks are characterised by the highest cross-linking densities, 135 g/mol for the PMHS(D_4_^Vi^) system and 118 g/mol for PMHS(Q(M^Vi^)_4_), suggesting that the Ag NPs are predominantly deposited on the carrier surface and do not limit the thermal cross-linking process. Furthermore, the loss of Si-H groups in these systems was minimal, amounting to 15% and 9% for the networks cross-linked with D_4_^Vi^ and Q(M^Vi^)_4_, respectively. The improvement in the thermal properties of the PMHS(Q(M^Vi^)_4_)Ag system is likely influenced by the minimal loss of Si-H groups and the presence of Ag NPs exclusively on the carrier surface, which may slightly delay the thermal degradation process of this polymer network.

The systems under investigation exhibit a two-stage thermal decomposition process, as indicated by the presence of two minima in their corresponding DTG curves. An exception is the PMHS(^Vi^MM^Vi^)Ag sample, for which the DTG curve shows three distinct minima of different intensity. The maximum temperatures corresponding to each step of thermal decomposition are consolidated in Table 1. Considering previous research on polysiloxane networks and the findings of the MS investigations of the samples described in the subsequent part of this study, the decomposition steps at temperatures around 500 °C can be attributed to redistribution reactions that involve Si-containing bonds (Si-O/Si-C, Si-H/Si-O, Si-O/Si-O), which result in the release of volatile silicon compounds. The next stage of decomposition, called the mineralisation stage (ceramisation), takes place at a temperature of about 700 °C and is associated with the release of gaseous hydrocarbons and H_2_ due to radical processes involving Si-C and C-H or Si-H bonds [35,36,37]. In the PMHS(^Vi^MM^Vi^)Ag sample at a temperature of about 500 °C, two minima are recorded on the DTG curve at 481 and 537 °C, respectively, indicating a two-stage redistribution of bonds at the silicon atom.

The presence of Ag NPs in the polysiloxane matrix affects the temperature of maximum mass loss in the individual stages of decomposition of the systems studied (Table 1). The greatest differences are observable for the stage of redistribution of bonds at the silicon atom. In the V_3_(^H^MM^H^) system, the presence of Ag NPs increases T_max_ 30–650 °C by 22 °C, while in the V_3_(D_4_^H^) sample, the situation is reversed, and T_max_ 30–650 °C decreases by 21 °C. In the remaining systems, the differences do not exceed 10 °C. The influence of Ag NPs on the mineralisation stage is relatively small. The differences in the percentage of mass loss and T_max_ 650–1000 °C for the initial systems and those containing silver do not exceed 1.5% and 10 °C, respectively.

**Figure 3:**
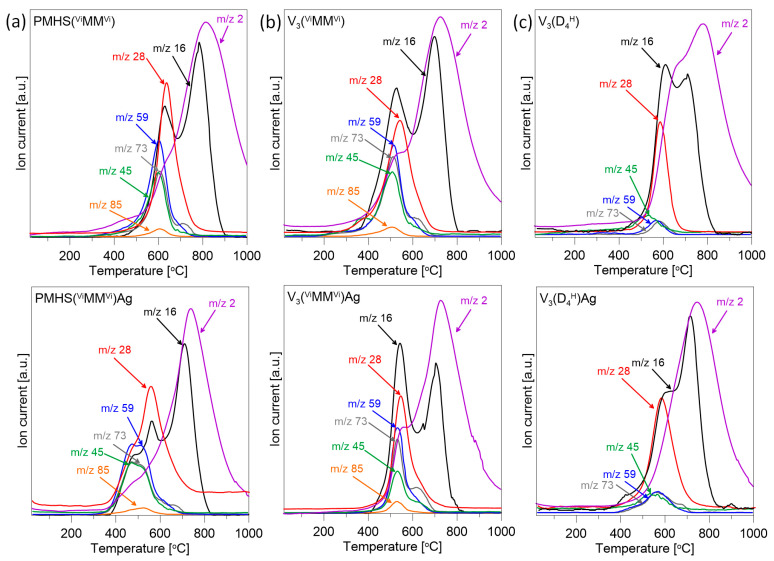
The ion current of the major gaseous species released from selected initial networks and polysiloxane/Ag nanocomposites during thermal pyrolysis: (**a**) PMHS(^Vi^MM^Vi^) and PMHS(^Vi^MM^Vi^)/Ag, (**b**) V_3_(^H^MM^H^) and V_3_(^H^MM^H^)/Ag, (**c**) V_3_(D_4_^H^) and V_3_(D_4_^H^)/Ag.

Mass spectrometry was used to analyse the gases released from the studied samples during the pyrolysis process. The relationships observed between the MS results and the TG profiles help to provide some suggestions about weight-loss mechanisms. Figure 3 presents the results of MS analysis for three selected polysiloxane networks and their nanocomposites with Ag NPs. The TG/DTG curves of these samples indicated the greatest differences in the thermal properties of the materials studied. The MS results for the remaining initial polysiloxane networks and their nanocomposites did not show significant differences. For better clarity, only the ion current (IC) curves corresponding to the most intensive *m*/*z* lines of decomposition products released during pyrolysis are shown in Figure 3.

In all the samples analysed, *m/z* lines equal to 2, 16, 28, 45, 59, 73 were recorded. Moreover, in the polymer networks in which the linear ^H^MM^H^/^Vi^MM^Vi^ was used as the cross-linking agent, an *m/z* line equal to 85 was present. The volatile silane species CH_3_SiH_2_^+^ (*m/z* = 45), (CH_3_)_2_SiH^+^ (*m/z* = 59), (CH_3_)_3_Si^+^ (*m*/*z* = 73), (CH_3_)_2_Si(CH=CH_2_)^+^ (*m*/*z* = 85), and ethene or ethane (*m*/*z* = 28) were released during the bond redistribution reaction at the silicon atom. Hydrogen (*m*/*z* = 2) and methane (*m*/*z* = 16) were also detected in this temperature range, but their presence is mainly related to the fragmentation of the released silicon compounds [37,38,39,40,41,42]. The bond redistribution reactions at the silicon atom, which lead to the release of volatile silicon compounds, have been described in detail in the works [37,41].

The IC profiles obtained for the initial networks prepared from linear disiloxane show that the deterioration in the thermal stability of V_3_(^H^MM^H^) in relation to PMHS(^Vi^MM^Vi^) is related to the earlier initiation of the redistribution of bonds at the silicon atoms (Figure 3a,b). The maximum of all the *m*/*z* lines assigned to volatile silicon compounds (*m*/*z* = 45, 59, 73, 85) in the V_3_(^H^MM^H^) system is located at ~500 °C, while in PMHS(^Vi^MM^Vi^) it is located at ~600 °C. Furthermore, in the V_3_(^H^MM^H^) sample, the intensity of the *m*/*z* = 28 line, which is related to the evolution of ethene or ethane, already increases slightly from a temperature of ~300 °C. The release of ethene or ethane is related to the cleavage of Si-C bonds in Si-CH_2_-CH_2_-Si bridges formed during the cross-linking reaction and/or the decomposition of vinyl groups. Previous FTIR studies of these polysiloxane networks showed that in the V_3_(^H^MM^H^) sample after the hydrosilylation process, unreacted Si-CH=CH_2_ groups remained [25], which are not present in PMHS(^Vi^MM^Vi^) and may be responsible for the appearance of the line *m*/*z* = 28 at lower temperatures.

The presence of Ag NPs in most of the systems studied causes the initiation of the silicon bond redistribution process at lower temperatures than in the initial polymer networks (Figure 3a,c). This is most visible in the PMHS(^Vi^MM^Vi^) Ag sample, in which the silicon bond redistribution process takes place in two stages (Figure 3a), the first at lower temperatures than in the initial network without metal. This is indicated by the curves *m*/*z* = 45, 59 and 73, which have two maxima of similar intensity. On the other hand, in the V_3_(^H^MM^H^)Ag sample, an increase in the starting temperature of the release of volatile silicon and ethene/ethane compounds is observable. In this sample, the presence of Ag NPs probably initially limits the initiation of the silicon bond redistribution stage. It is also visible that the earlier decomposition of the vinyl groups present in the polymer matrix was eliminated, as no release of ethene or ethane was observed at a temperature of ~300 °C (Figure 3b). This may be due to the arrangement of silver nanoparticles not only on the carrier surface but also inside the network, which may initially constitute a physical obstacle to the release of volatile decomposition products, improving the thermal stability of the system.

**Figure 4:**
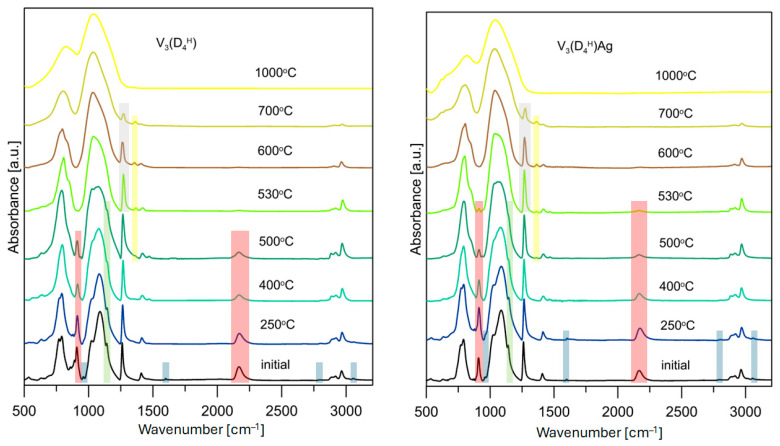
Pyrolytic transformation of polysiloxane networks and polysiloxane/Ag nanocomposites to SiOC and Ag/SiOC materials monitored by FTIR spectroscopy.

To better understand the effect of the presence of Ag NPs on the degradation mechanism of the polysiloxane networks studied, samples V_3_(D_4_^H^) and V_3_(D_4_^H^)/Ag, which were characterised by the largest differences in the course of the TG curves, were subjected to pyrolysis at temperatures selected based on the DTG curves. Figure 4 shows the transformation of the polysiloxane networks studied and their Ag nanocomposites into silicon oxycarbide ceramics and Ag/SiOC nanocomposites. The detailed course of the polysiloxane networks pyrolysis process, obtained as a result of the hydrosilylation reaction [31,34,43] and the sol-gel method [44,45], has already been described in the literature. For this reason, in this work, attention is mainly focused on the effect of the presence of Ag NPs on the pyrolysis process.

In the FTIR spectra of the V_3_(D_4_^H^) polymer network, despite the excess of Si-H groups used in the hydrosilylation reaction, bands corresponding to vinyl groups are present at 3056, 3016, 1598, and 960 cm^–1^ (marked blue in Figure 4). The intensity of these bands is low, and they are absent in the spectrum recorded for the pyrolysis product of the V_3_(D_4_^H^) sample obtained at 250 °C, but still present at this temperature in the V_3_(D_4_^H^)Ag sample. The earlier disappearance of the bands corresponding to vinyl groups in the initial polymer network is likely related to the thermal cross-linking process within the polysiloxane network, involving Si-CH=CH_2_ and Si-H groups, resulting in the formation of Si-CH_2_-CH_2_-Si bridges. This cross-linking process does not produce byproducts, which is why no mass loss was recorded on the TG curve. This thermal cross-linking has also been proposed in other polysiloxane networks where the unreacted Si-CH=CH_2_ and Si-H groups remained after the hydrosilylation reaction [46]. The results obtained indicate that Ag NPs in the polysiloxane matrix act as a physical barrier, limiting the thermal cross-linking process and thus reducing the thermal stability of the V_3_(D_4_^H^)Ag system.

The characteristic band for the Si-CH_2_-CH_2_-Si formed during the hydrosilylation reaction is located at 1138 cm^−1^ (marked green in Figure 4), and the strong bands in a range of 1031–1097 cm^−1^, which are attributed to the asymmetric stretching vibrations of the Si-O-Si groups. During pyrolysis, a decrease in the intensity of this band is observed. It remains present in the FTIR spectra of the analysed samples at 500 °C, while up to 530 °C, the ethylene bridges are completely decomposed. However, it should be noted that in the FTIR spectrum at 500 °C for the V_3_(D_4_^H^)Ag system, at 1138 cm^−1^, there is only a slight inflection compared to the distinct band for the V_3_(D_4_^H^) sample. This indicates a lower content of ethylene bridges at 500 °C, and their faster decomposition in the V_3_(D_4_^H^)Ag system.

The bands corresponding to the vibrations of the Si-H groups are located at 910 and 2160 cm^−1^ (marked red in Figure 4). The intensity of these bands decreases in the analysed samples as the pyrolysis temperature increases. In the V_3_(D_4_^H^) sample, a rapid disappearance of these bands is observed between 500 and 530 °C. It should also be noted that at 500 °C, a band appears at 1359 cm^−1^, which is associated with the formation of Si-CH_2_-Si bridges (marked yellow in Figure 4). Methylene bridges are formed as a result of reactions between Si-CH_3_ and Si-CH_3_ or between Si-CH_3_ and Si-H, releasing methane and hydrogen as byproducts. Between 500 and 530 °C, an increase in the intensity of the band is observed at 1359 cm^−1^, suggesting that Si-H groups play a significant role in the formation of new Si-CH_2_-Si bridges within this temperature range. In the V_3_(D_4_^H^)Ag sample, the intensity of the bands corresponding to the Si-H groups is significantly lower at 500 °C compared to the sample without metal. However, these bands are still present in the FTIR spectra at 530 °C. The bands originating from the vibrations of the Si-CH_2_-Si bridges also appear at 500 °C, but their intensity increases only slightly in the spectra recorded at 530 °C. It should also be noted that the intensity of the bands corresponding to the vibrations of Si-CH_3_ groups, such as the one at 1260 cm^−1^, does not change up to 500 °C in both of the systems studied (marked grey in Figure 4). Above this temperature, a faster loss of the Si-CH_3_ groups is observed in the V_3_(D_4_^H^) system. The results obtained suggest that the Si-H and Si-CH_3_ groups in the metal-containing sample participate to a lesser extent in the formation of Si-CH_2_-Si connections, which may be related to the restricted access between these groups due to the presence of Ag NPs.

**Figure 5:**
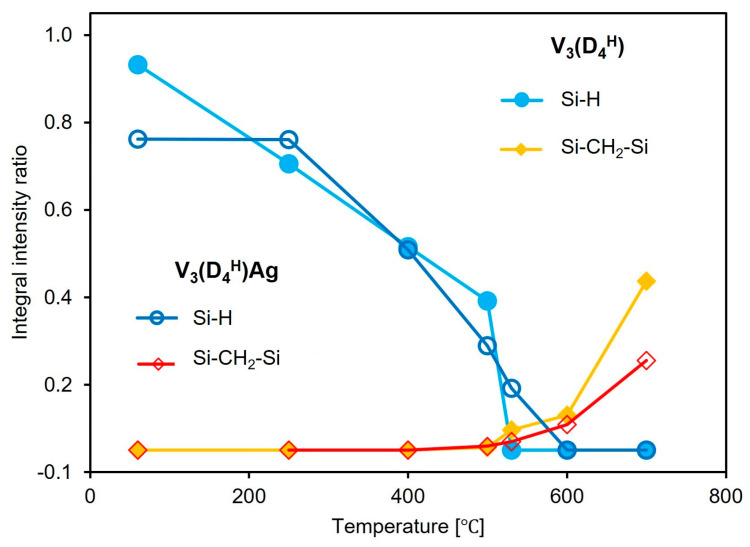
Evolution of Si-H and Si-CH_2_-Si bands with increasing pyrolysis temperature in V_3_(D_4_^H^) and V_3_(D_4_^H^)Ag samples (the integral intensity ratios refer to the Si-H/Si-CH_3_ and Si-CH_2_-Si/Si-CH_3_).

To determine the contribution of the bands associated with Si-H groups and Si-CH_2_-Si bridges during pyrolysis, and to track their evolution with increasing pyrolysis temperature, a quantitative analysis of the FTIR spectra was performed. The integral intensity ratios of the Si-H/Si-CH_3_ and Si-CH_2_-Si/Si-CH_3_ bands obtained at different pyrolysis temperatures (Section 2.2) are presented in Figure 5. The band at 1260 cm^−1^, corresponding to the vibrations of the Si-CH_3_ groups, was used as a reference band, as its shape and intensity remain constant up to 500 °C. Quantitative analysis of the FTIR spectra revealed that in the V_3_(D_4_^H^) sample, Si-H groups disappear from the polysiloxane network at temperatures below 250 °C, whereas in the metal-containing sample, the number of Si-H groups remains unchanged. This confirms the previous conclusion that in the sample without metal, thermal cross-linking occurs, involving the Si-H and Si-CH=CH_2_ groups. The presence of Ag NPs in the V_3_(D_4_^H^)Ag sample prevents the occurrence of additional hydrosilylation reactions involving these functional groups. The loss of Si-H groups with increasing temperature in the initial sample occurs more slowly than in the sample containing metal, until it accelerates sharply between 500 and 530 °C. Such a rapid loss of Si-H groups is not observable in the V_3_(D_4_^H^)Ag system. The rapid loss of these groups in the V_3_(D_4_^H^) sample in this temperature range is associated with a significantly greater increase in the formation of Si-CH_2_-Si bridges in the V_3_(D_4_^H^) system compared to V_3_(D_4_^H^)Ag. It should be noted that above 500 °C, the FTIR spectra show a depletion of Si-CH_3_ groups, which affects the ratio of the integral intensities of the analysed bands. However, there is no doubt that a higher number of Si-CH_2_-Si bridges is observed in the system without metal compared to the metal-containing sample. The results suggest that silver nanoparticles limit reactions between the Si-H and Si-CH_3_ groups, as well as between the Si-CH_3_ and Si-CH_3_ groups, thus reducing the number of Si-CH_2_-Si linkages formed.

**Figure 6:**
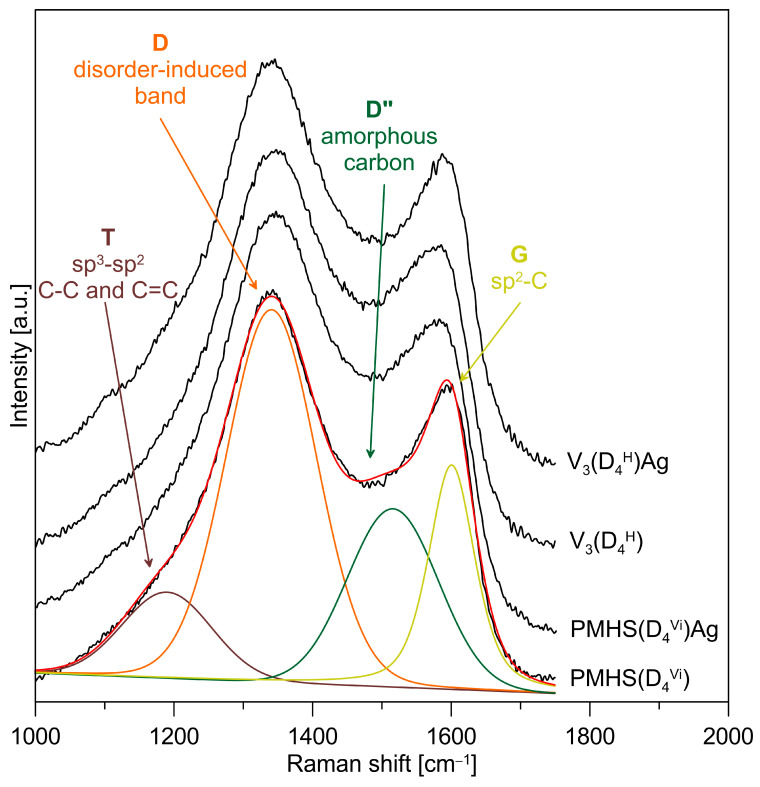
Raman spectra of the SiOC and Ag/SiOC nanocomposite materials obtained from polymer precursors cross-linked with a cyclic cross-linking agent. Deconvoluted spectrum of the PMHS(D_4_^Vi^) and assignments of the component bands.

The FTIR spectrum of the pyrolysis product at 1000 °C confirms the presence of Si–O and Si–C bonds typical for SiOC materials, and also confirms the receipt of silicon oxycarbides (Figure 4). The SiOC and Ag/SiOC nanocomposite materials were subjected to Raman investigation to verify if the free-carbon phase was present in the studied systems. Figure 6 shows the Raman spectra for selected studied materials along with the deconvoluted spectrum of the PMHS(D_4_^Vi^) and the assignments of the component bands. The two most intense and important bands are the so-called disorder-induced D band at ~1350 cm^−1^ and the G band at ~1600 cm^−1^ due to in-plane bond stretching of sp^2^ carbon. Furthermore, T bands (shoulder at ~1200 cm^−1^ assigned to the presence of sp^3^-sp^2^ C-C and C=C bonds) and D″ bands (at ~1500 cm^−1^ corresponding to the fraction of amorphous carbon contained in the samples) are also present in the spectra. The presence of T and D″ bands suggests a somewhat disordered state, which could be caused by factors such as the presence of edges in the graphene layers, the deviation from planarity of graphene layers, the presence of pores, or carbon atoms exhibiting sp^3^ hybridisation [47,48]. The recorded spectra for all the studied SiOC materials contain four bands characteristic for graphitic-like and amorphous carbon, which occur in silicon oxycarbides. The type of polymer precursor used and the presence of metal do not cause significant differences in the recorded Raman spectra.

The ratio of the integral intensity of the D and G bands, obtained after the deconvolution process of the Raman spectra, was used to calculate the crystalline size of the graphite domains (L_a_) (Section 2.2). The results obtained, presented in Table 2, show that the L_a_ is in the range of 1.4–1.8 nm and is in a similar range to those of the SiOC materials obtained from polymer precursors prepared by the sol-gel method (1.6 nm) [45]. The presence of Ag NPs in the polysiloxane matrix does not significantly affect L_a_; the L_a_ in Ag/SiOC nanocomposites is in the range of 1.4–1.7 nm. The largest difference in L_a_ was observed in samples with and without metal for the V_3_(D_4_^H^) system, in which the presence of Ag NPs had the greatest effect on the pyrolysis process among the samples studied.

**Figure 7:**
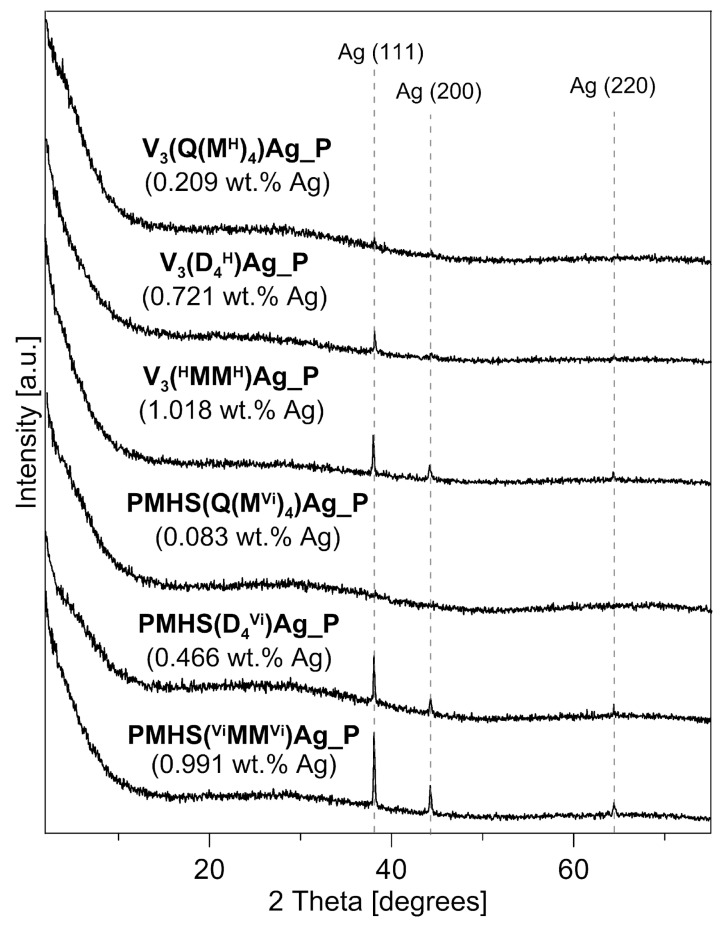
X-ray diffraction patterns of the polysiloxane/Ag nanocomposites studied after pyrolysis at 1000 °C. The silver content in the samples after pyrolysis is given in brackets.

The XRD patterns of the polysiloxane/Ag nanocomposites after pyrolysis at 1000 °C are presented in Figure 7. In all of the pyrolysed samples, a broad peak of low intensity centred at 2 theta = ~25° is present. This broad peak is associated with the presence of a lamellar structure, such as graphitic carbon, which occurs in silicon oxycarbides [2,19,20]. Such structures also occur in the studied samples, as shown by the discussed Raman investigations. Apart from the broad reflection, distinct reflections at 2 theta angle values of 38.1°, 44.3°, and less, resolved at 64.4°, were recorded in the diffraction patterns of most of the nanocomposites, with the exception of the sample of PMHS(Q(M^Vi^)_4_)Ag_P. These reflections can be attributed to the metallic crystalline phase of silver, specifically the Ag (111), Ag (200), and Ag (220) crystallographic planes, respectively. The silver reflections in the samples after pyrolysis are more intense and better resolved than those of the corresponding initial polysiloxane/Ag nanocomposites before pyrolysis [25]. The higher intensity of the recorded reflections is primarily related to the higher amount of silver in the samples after pyrolysis. As described earlier, during the pyrolysis of polysiloxane networks, the mass loss observed on the TG curves is related to the release of low-molecular silicon compounds, gaseous hydrocarbons, and hydrogen. The amount of silver in the sample does not change, but its percentage content increases, as a result of mass loss during the transformation of the polysiloxane matrix into a ceramic matrix. Figure 7 shows the Ag content in the sample after pyrolysis at 1000 °C (values in brackets). The Ag content in the analysed samples is in the range of 0.083–1.018 wt.%. In the sample with the lowest metal content, i.e., PMHS(Q(M^Vi^)_4_)Ag_P, which contained only 0.083 wt.% Ag, no reflections from the metallic phase were recorded. In the sample with the second-lowest result, i.e., V_3_(Q(M^H^)_4_)Ag_P (0.209 wt.% Ag), a reflection of very low intensity was recorded.

The higher intensity of reflections from metallic silver can also be associated with an increase in the size of Ag crystallites during pyrolysis. This is especially visible in the samples cross-linked with a cyclic agent. In the initial systems, the metal content was 0.393 and 0.564 wt.% for PMHS(D_4_^Vi^)Ag and V_3_(D_4_^H^)Ag, respectively, and the diffractograms of these systems did not have any reflections of Ag, indicating their small size and large dispersion on the support surface [25]. The samples after pyrolysis are characterised by an increase in the Ag content of about 20%, and their diffractograms show clear reflections from the metallic phase. An increase in the size of metal crystallites after the pyrolysis process at 1000 °C has also been observed in polysiloxane networks with Pt NPs [32].

**Figure 8:**
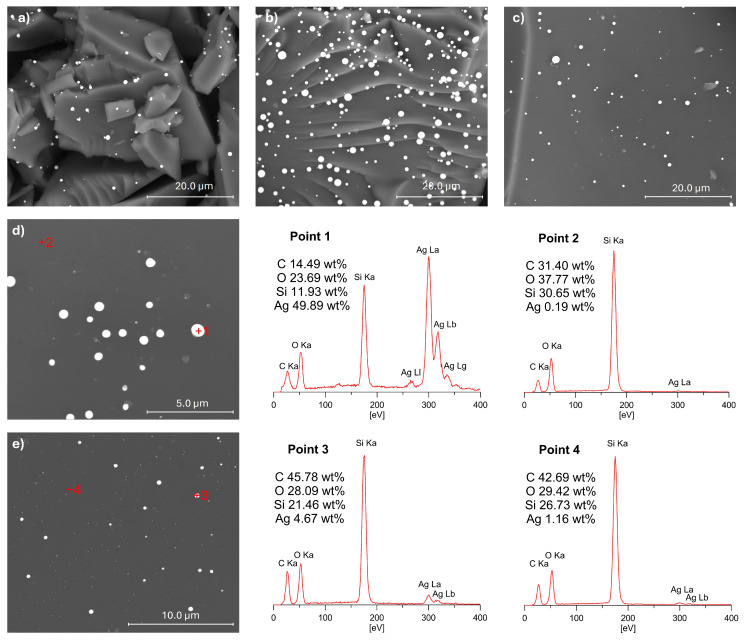
SEM images of selected samples (**a**) PMHS(^Vi^MM^Vi^)Ag_P, (**b**) PMHS(D_4_^Vi^)Ag_P, (**c**) V_3_(Q(M^H^)_4_)Ag_P, (**d**) PMHS(D_4_^Vi^)Ag_P, and (**e**) V_3_(D_4_^H^)Ag_P, with the results of EDX analysis shown for the points marked by red ‘+’ symbols in (**d**,**e**).

To investigate the distribution of Ag NPs on the surface of the SiOC materials, SEM imaging was performed. SEM images of selected samples after pyrolysis at 1000 °C are presented in Figure 8. The observations confirmed the presence of Ag NPs on the surface of all the analysed SiOC materials, with a uniform distribution across the substrate. SEM analysis revealed that after pyrolysis, the Ag particles appear larger than in the initial polysiloxane networks, forming spherical agglomerates [25]. These findings align with the XRD results, which indicated an increase in Ag crystallite size during the thermal transformation of polysiloxane networks into SiOC material. The size of the silver particles on the ceramic matrix surface varied, with larger Ag particles around 1 μm in diameter, as well as smaller particles measuring several hundred nanometres, and very fine particles visible as bright dots on the analysed substrate surfaces.

EDX analyses were performed at various points on the surfaces of the samples studied, with selected results presented in Figure 8. The EDX data revealed a high concentration of silver at bright spots (Point 1), indicative of significant Ag accumulation. Notably, even in grey regions (Points 2 and 4), where no visible silver particles were observed, the silver content remained at around 1 wt% or lower. These findings suggest that not all of the silver particles on the SiOC support surface agglomerated; some likely retained nanoscale dimensions, similar to their size in the prepyrolysis samples [25]. This indicates a partial retention of nanoscale dispersion despite high-temperature processing.

## 4. Conclusions

This study investigated the effect of Ag NPs on the thermal properties of polysiloxane networks with different network structures and distributions of Si–H groups. Polysiloxane matrices were obtained from two types of polymers, i.e., PMHS and V_3_, and three cross-linking agents that differed in molecular structure (linear, linear branched, and cyclic) and functionality (difunctional and tetrafunctional). Thermogravimetric analysis revealed that the presence of Ag NPs generally degraded the thermal properties of most of the studied systems. In systems derived from the V_3_ polymer, which exhibited lower cross-linking densities, the influence of Ag NPs was more pronounced compared to their PMHS counterparts. The presence of metal in the samples affected the pyrolysis process of the systems analysed, limiting thermal cross-linking and the formation of Si–CH_2_–Si bridges, resulting in a reduced ceramic yield of the studied materials. Pyrolysis of polysiloxane/Ag nanocomposites conducted under appropriate conditions (1000 °C, Ar atmosphere) leads to the formation of Ag/SiOC nanocomposites. Despite the deterioration of the thermal properties of the Ag NP-containing systems, Ag/SiOC materials were obtained with ceramic residues in the range of 43–84%. Raman spectroscopy analysis of SiOC and Ag/SiOC materials confirmed the presence of graphitic-like and amorphous free-carbon phases, and further showed that the presence of silver nanoparticles did not significantly influence the size of graphite crystallites (L_a_) in the studied materials. XRD analysis of the Ag/SiOC materials demonstrated an increase in the intensity of reflections from the metallic silver phase, which is attributed both to the increased silver content resulting from mass loss in the polysiloxane matrix during pyrolysis, and to the growth in the silver crystallite size, especially in samples that were cross-linked using the cyclic cross-linking agent. The SEM and EDX analyses demonstrate that Ag NPs are uniformly distributed on the surface of SiOC materials, with some retention of nanoscale dispersion despite the high-temperature pyrolysis, while other particles agglomerate into larger spherical clusters, confirming the partial stability of the nanoscale structure during thermal processing.

## Figures and Tables

**Table 1:** Values of maximum temperatures and respective mass losses in each decomposition step obtained from the TG/DTG curves.

Sample	T_5%_ [°C]	T_max_ 30–650 °C [°C]	Mass Loss 30–650 °C [%]	T_max_ 650–1000 °C [°C]	Mass Loss 650–1000 °C [%]	Residue at 1000 °C [%]
PMHS(^Vi^MM^Vi^)	468	538	37.7	702	5.5	56.8
PMHS(^Vi^MM^Vi^)Ag	424	481, 537	39.7 (19.1, 20.6)	711	5.7	54.6
PMHS(D_4_^Vi^)	590	584	10.2	702	5.7	84.1
PMHS(D_4_^Vi^)Ag	584	577	9.8	710	5.8	84.4
PMHS(Q(M^Vi^)_4_)	517	552	16.9	702	6.8	76.3
PMHS(Q(M^Vi^)_4_)Ag	536	557	15.2	697	7.3	77.5
V_3_(^H^MM^H^)	425	515	36.5	692	6.5	57.0
V_3_(^H^MM^H^)Ag	483	537	52.5	699	4.1	43.4
V_3_(D_4_^H^)	611	588	7.9	707	5.3	86.8
V_3_(D_4_^H^)Ag	548	567	14.9	706	6.9	78.2
V_3_(Q(M^H^)_4_)	537	553	14.7	696	8.3	77.0
V_3_(Q(M^H^)_4_)Ag	521	551	19.6	707	8.1	72.4

**Table 2:** The crystalline size of the graphite domains in the studied materials pyrolysed at 1000 °C.

Sample	Cluster Size L_a_ [nm] ^a^	Sample	Cluster Size L_a_ [nm] ^a^
PMHS(^Vi^MM^Vi^)	1.4	PMHS(^Vi^MM^Vi^)Ag	1.4
PMHS(D_4_^Vi^)	1.7	PMHS(D_4_^Vi^)Ag	1.7
PMHS(Q(M^Vi^)_4_)	1.4	PMHS(Q(M^Vi^)_4_)Ag	1.5
V_3_(^H^MM^H^)	1.5	V_3_(^H^MM^H^)Ag	1.4
V_3_(D_4_^H^)	1.8	V_3_(D_4_^H^)Ag	1.4
V_3_(Q(M^H^)_4_)	1.6	V_3_(Q(M^H^)_4_)Ag	1.6

^a^ C(λ_L_) = 4.96.

## Data Availability

The data presented in this study are available on request from the corresponding author.
